# Effect of Inlet Air Temperature and Quinoa Starch/Gum Arabic Ratio on Nanoencapsulation of Bioactive Compounds from Andean Potato Cultivars by Spray-Drying

**DOI:** 10.3390/molecules28237875

**Published:** 2023-11-30

**Authors:** Carlos A. Ligarda-Samanez, David Choque-Quispe, Elibet Moscoso-Moscoso, Lizeth M. Flores Pozo, Betsy S. Ramos-Pacheco, Henry Palomino-Rincón, Rodrigo J. Guzmán Gutiérrez, Diego E. Peralta-Guevara

**Affiliations:** 1 Food Nanotechnology Research Laboratory, Universidad Nacional José María Arguedas, Andahuaylas 03701, Peru; elibetmm22@gmail.com; 2 Nutraceuticals and Biomaterials Research Group, Universidad Nacional José María Arguedas, Andahuaylas 03701, Peru; dchoque@unajma.edu.pe (D.C.-Q.); bsramos@unajma.edu.pe (B.S.R.-P.); hpalomino@unajma.edu.pe (H.P.-R.); 1008820181@unajma.edu.pe (R.J.G.G.); diepltagvra@gmail.com (D.E.P.-G.); 3 Research Group in the Development of Advanced Materials for Water and Food Treatment, Universidad Nacional José María Arguedas, Andahuaylas 03701, Peru; 4 Agroindustrial Engineering, Universidad Nacional José María Arguedas, Andahuaylas 03701, Peru; 1011220181@unajma.edu.pe; 5 Water and Food Treatment Materials Research Laboratory, Universidad Nacional José María Arguedas, Andahuaylas 03701, Peru

**Keywords:** nanocapsules, phenolic extracts, wall materials, quinoa starch, phenolic compounds, antioxidant capacity, factorial design

## Abstract

Nanoencapsulation of native potato bioactive compounds by spray-drying improves their stability and bioavailability. The joint effect of the inlet temperature and the ratio of the encapsulant (quinoa starch/gum arabic) on the properties of the nanocapsules is unknown. The purpose of this study was to determine the best conditions for the nanoencapsulation of these compounds. The effects of two inlet temperatures (96 and 116 °C) and two ratios of the encapsulant (15 and 25% *w*/*v*) were evaluated using a factorial design during the spray-drying of native potato phenolic extracts. During the study, measurements of phenolic compounds, flavonoids, anthocyanins, antioxidant capacity, and various physical and structural properties were carried out. Higher inlet temperatures increased bioactive compounds and antioxidant capacity. However, a higher concentration of the encapsulant caused the dilution of polyphenols and anthocyanins. Instrumental analyses confirmed the effective encapsulation of the nuclei in the wall materials. Both factors, inlet temperature, and the encapsulant ratio, reduced the nanocapsules’ humidity and water activity. Finally, the ideal conditions for the nanoencapsulation of native potato bioactive compounds were determined to be an inlet temperature of 116 °C and an encapsulant ratio of 15% *w*/*v*. The nanocapsules obtained show potential for application in the food industry.

## 1. Introduction

Nanoencapsulation of bioactive compounds is a technique that allows for the protection and controlled release of these compounds, improving their stability and bioavailability [1]. Native potato bioactive compounds, such as polyphenols and carotenoids, have demonstrated antioxidant, anti-inflammatory, and chemopreventive effects [2,3,4]. However, they present sensitivity to environmental factors and low solubility in aqueous media, which limits their application [5,6]. Nanoencapsulation is a promising strategy to overcome these technological limitations [7]. Among the encapsulating materials, quinoa starch has recently emerged as a modern and sustainable matrix due to its suitable physicochemical properties, which could be used in the food and pharmaceutical industries [8,9,10,11,12,13].

There are several methods for the encapsulation of bioactive compounds. Physical methods include emulsification, which encapsulates lipophilic compounds in oil-in-water (O/W) emulsions; ionic gelation and extrusion, which form particles by ionic cross-linking of polysaccharides; spray-drying, widely used because of its scalability; freeze-drying, which generates highly stable and porous particles; and electrospinning, which produces nanocapsules by applying high voltage to a polymer solution [14,15,16,17,18]. Chemical methods include interface polymerization, molecular cocrystallization, and inclusion in cyclodextrins [19,20]. Recently, biodegradable polymeric systems with bioactive substances are being explored by nanoprecipitation, layer-by-layer assembly, and functional group conjugation [21,22,23]. The selection of the encapsulation method depends on the characteristics of the active material and the desired application. It is critical to identify those conditions that guarantee stability and controlled release of the encapsulated compounds [24,25].

Among the nanoencapsulation techniques, spray-drying is widely used at the industrial level because of its scalability and low cost [26]. However, the process conditions can affect the characteristics of the obtained nanocapsules [27]. Two critical parameters are the inlet temperature of the equipment and the percentage of the encapsulant material [28,29]. The inlet temperature impacts the rate of moisture evaporation, influencing particle morphology and size [30]. High temperatures favor rapid evaporation, while low temperatures promote more uniform encapsulation [31]. On the other hand, the nature and concentration of the encapsulating material affect properties such as encapsulation efficiency, system stability, and release control [32,33]. Starch, proteins, maltodextrin, and gum arabic are employed to nanoencapsulate phenolic compounds and carotenoids [13,34,35,36,37].

Few studies systematically evaluate the combined effect of inlet temperature and percentage of quinoa starch/gum arabic encapsulant on the properties of nanocapsules containing bioactive compounds from native potatoes. Investigating the interaction between these factors would allow for the optimal conditions to maximize the stability and bioaccessibility of these compounds to be determined. The objective of this work was to study the effect of the inlet temperature (96 and 116 °C) and the percentage of encapsulant (15% and 25%) on the spray-drying nanoencapsulation process of bioactive compounds extracted from native potatoes. The response variables evaluated were phenolic compounds, flavonoids, anthocyanins, antioxidant capacity, yield, moisture, water activity, and particle size, in addition to a modern instrumental characterization and release of phenolic compounds. The study is expected to generate knowledge about the interactions between the studied variables and the properties of the nanocapsules, determining optimal conditions for the encapsulation of thermosensitive compounds of native potatoes. This work provides knowledge to maximize the stability and bioavailability of bioactive compounds from undervalued Andean crops, having a positive impact at the local and regional level in Peru. It will also allow for the technological problems associated with the nanoencapsulation of thermosensitive compounds globally to be solved.

## 2. Results and Discussion

### 2.1. Characterization and Selection of the Best Native Potato

Table 1 shows the physicochemical properties of the native potatoes studied. The phenolic compounds ranged from 2.84 to 3.90 mg gallic acid equivalent per gram (GAE/g), being similar to that reported in native potato clones in Peru (4.57–6.72 mg GAE/g) and Chile (1.92–18.64 mg GAE/g) [4,38]. Higher levels of polyphenols are associated with higher antioxidant activity due to their ability to neutralize free radicals [39].

The anthocyanin content ranged from 135.53 to 401.22 mg cyanidin-3-glucoside per gram (C3G/g), being higher than that reported in native potato clones in Peru (2.53–7.79 mg C3G/g). Anthocyanins are antioxidant pigments that confer a purple color to tubers [4,40].

The flavonoids ranged from 0.46 to 0.74 mg quercetin equivalent per gram (QE/g), which was lower than values reported in native potato clones in Peru (1.68–3.00 mg QE/g) [4] and colored potatoes in Bolivia (19 mg QE/g) [41]. On the other hand, these values were higher than those recorded in colored potatoes from New Zealand (0.03 mg QE/g) [42]. Quercetin is the primary antioxidant flavonoid present in potatoes; flavonoids protect against oxidative stress by donating hydrogen atoms [4,43].

Antioxidant capacity measured by 2,2-Diphenyl-1-Picrylhydrazyl (DPPH) ranged from 4.35 to 23.43 μmol Trolox equivalent per gram (TE/g) and by 2,2′-Azinobis-3-ethyl-benzo-thiazoline-6-sulfonic acid (ABTS) from 9.99 to 19.22 μmol TE/g; both were comparable to values in native potato clones and positively correlated with total phenols [4,41]. Higher antioxidant capacity could improve stability and provide health benefits [44].

As for color, lightness (L*) ranged from 13.55–22.15, and chroma a (a*) and chroma b (b*) values were analogous to those of pigmented potatoes, being linked to anthocyanins that confer reddish and purple tones [4,45]. Moisture, which is associated with a longer shelf life, fluctuated between 75.92 and 76.73%. Water activity (Aw), which influences stability and shelf life, was between 0.82 and 0.84, slightly exceeding that of other tubers, preventing microbial growth [45,46].

The variability in the abovementioned characteristics is attributed to genetic factors associated with the native potato variety, agroecological conditions, and cultivation techniques [4,45]. For this reason, due to the higher contents of bioactive compounds, the native potato variety Kulli papa was chosen to carry out nanoencapsulation using the spray-drying process.

### 2.2. Characterization of Matrices and Core

#### 2.2.1. Color, Particle Size, ζ-Potential, and SEM-EDS Analysis

**Figure 1:**
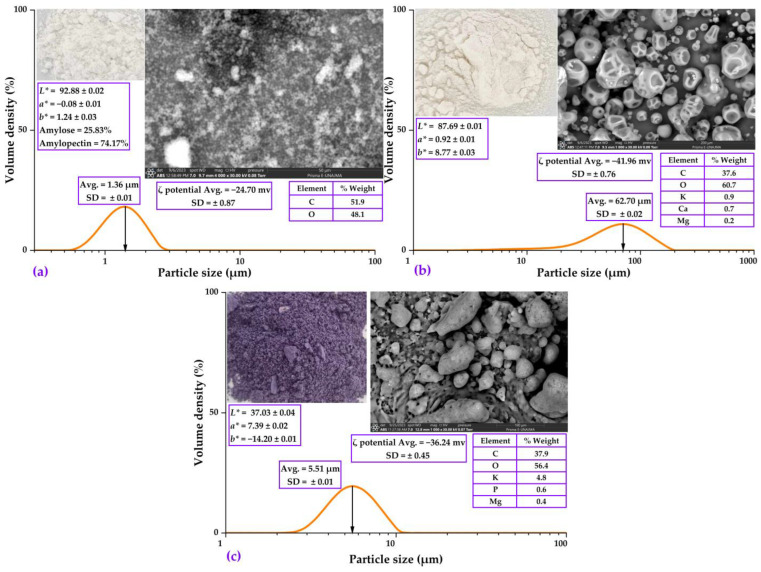
Characterization of matrices and core: (**a**) quinoa starch; (**b**) gum arabic; and (**c**) spray-dried ethanolic extract of native potato.

In Figure 1a, the characterization of quinoa starch obtained by hydro extraction is presented. This starch exhibits spherical particles with an average size of 1.36 µm, similar to that reported by Li (2018) [9], whose sizes ranged from 1 to 3 µm, indicating a small particle size compared to other starches, such as that of native potato (32.30 μm) [47]. The reduction in particle size during hydro extraction is due to the mechanical breakdown of starch granules, which generates smaller particles and improves functional properties such as solubility and swelling power [9,48,49,50]. Quinoa starch also shows slight stability (ζ potential = −24.70 mV) and is a white-colored powder, characteristic of native starches [32,33,47]. The amylose and amylopectin content are within the range reported for quinoa starch. The predominant presence of carbon and oxygen is consistent with the chemical composition of starch, mainly constituted by glucose molecules [9,51].

Figure 1b shows the characterization of gum arabic, which presented an average particle size of 62.70 μm and spherical shapes with indentations on the surface, higher than that reported for other natural gums, such as tara gum (3.12–8.53 μm) [32,47]. This is because gum arabic comes from a natural unprocessed exudate that generates larger aggregates [52]. Moreover, it showed good stability with ζ potential of −41.96 mV, attributed to the amphiphilic conformation of its polysaccharides that favors the formation of stable suspensions [53]. The white color and the composition, rich in carbon and oxygen, were expected for this natural polysaccharide [54]. The presence of minerals such as potassium, calcium, and magnesium agree with the composition reported for gum arabic from *Acacia senegal* [55].

Figure 1c presents the characterization of the phenolic extract of the selected native potato that was spray-dried; irregular shapes with an average particle size of 5.51 μm were observed, placing it within the microparticle range. Using the spray-drying technique, smaller particles are achieved compared to other techniques [56]. In addition, the atomized extract showed medium stability (ζ potential = −36.24 mV) and purple coloration characteristic of phenolic compounds [57]. The predominance of carbon and oxygen in its composition is consistent with its polyphenolic nature, in addition to the presence of minerals such as potassium, phosphorus, and magnesium, which agrees with the profile reported in native potatoes [4].

#### 2.2.2. Thermal Analysis

**Figure 2:**
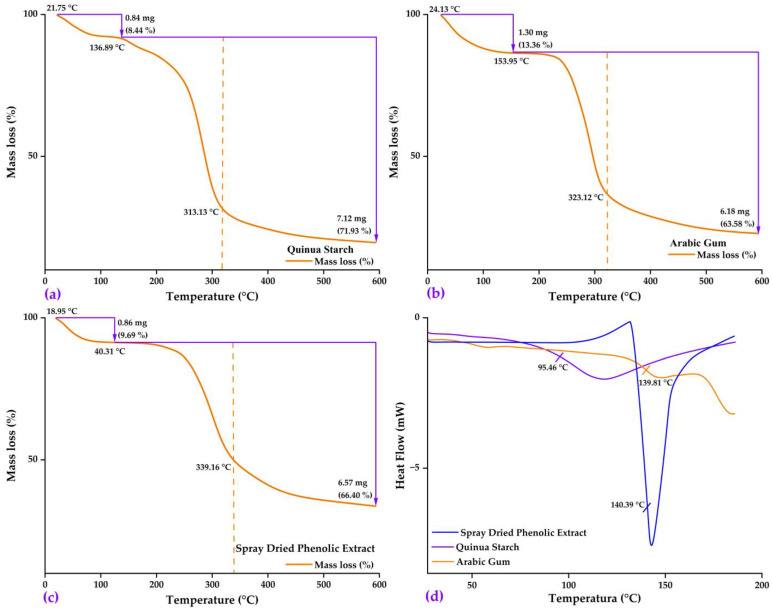
TG and DTA analysis in: (**a**) quinoa starch; (**b**) gum arabic; (**c**) spray-dried phenolic extract; and (**d**) DSC analysis in matrices and core.

Figure 2a shows the results of the thermal analysis of quinoa starch. The first event occurred between 21.75 and 136.89 °C, with a mass loss of 8.44%, corresponding to the evaporation of free water due to the hydrophilic nature of the functional groups of the polysaccharides present in this matrix. This agrees with studies such as that by Ligarda et al. (2022) [47]. A second event occurred between 136.89 and 600 °C (with a peak around 313.13 °C). This was associated with a mass loss of 71.93%, which is related to the thermal decomposition of starch, particularly the depolymerization of amylose and amylopectin [33].

A thermal analysis of gum arabic (Figure 2b) showed two main events: between 24.75 and 153.95 °C, a mass loss of 13.36% occurred due to the removal of absorbed water, which is consistent with the known hygroscopic character of gum [30]. Between 153.95 and 600 °C, with a peak at around 323.12 °C, a 63.58% mass loss associated with the thermal decomposition of biopolymers, including protein fractions and polysaccharides such as arabinogalactans, was recorded [4].

The thermal behavior of the spray-dried native potato phenolic extract (Figure 2c) evidenced two main mass loss events: between 18.95 and 140.31 °C a loss of 9.69% attributable to evaporation of water and volatile compounds, commensurate with the hygroscopic nature of the extract rich in phenols and sugars [4,58]; between 140.31 and 600 °C, with a peak around 339.16 °C, the loss was 66.40% due to thermal decomposition of the organic components of the extract [32].

Differential scanning calorimetry analysis (Figure 2d) determined glass transition temperatures (Tg) of 95.46 °C for quinoa starch, 139.81 °C for gum arabic and 140.39 °C for the phenolic extract of native potato, attributing the lower Tg of starch to its composition with a lower content of proteins and plasticizing lipids [11], while gum and phenolic extract presented higher Tg due to their structures with high molecular density and strong intermolecular interactions [4,30]. The results shown above provide valuable information on the thermal stability and composition of the matrices and core, being useful for applications involving heating or processing at high temperatures.

### 2.3. Nanoencapsulation of Phenolic Compounds

Table 2 shows the physicochemical properties studied in the nanocapsules obtained by spray-drying through a factorial design 2^2^. The results show that the nanocapsules presented variable amounts of bioactive compounds such as phenolic compounds (4.61–5.50 mg GAE/g), flavonoids (0.63–0.87 mg QE/g), and anthocyanins (0.91–2.32 mg C3G/g). The antioxidant capacity was also variable between 48.98 and 185.50 μmol ET/g, which may be related to differences in the bioactive compound content of the extracts [4,30].

The yields ranged between 61.99% and 66.64%, indicating that the spray-drying process was efficient in the encapsulation of the studied extracts [32]; the moisture ranged between 10.72% and 14.29%, and the water activity between 0.41 and 0.48; both properties were low, resulting in nanocapsules with good stability [33]. The particle size ranged from 289.57 to 478.70 nm, presenting a nanometer range suitable for the desired food and pharmaceutical applications [4,59].

**Figure 3:**
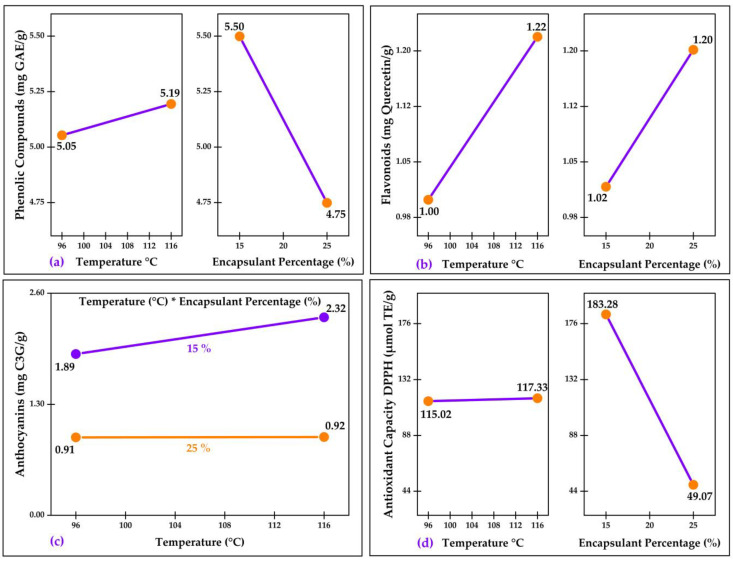
Effects and interactions in: (**a**) total phenolic compounds; (**b**) total flavonoids; (**c**) anthocyanins; and (**d**) antioxidant capacity. * Corresponds to the multiplication sign.

Figure 3a shows the effects of temperature and percentage of the encapsulant (*p* < 0.05) on the phenolic compounds. It can be observed that an increase in temperature leads to a significant increase in polyphenols. This phenomenon can be attributed to the almost instantaneous formation of a protective layer of gelatinized starch on the nanocapsules due to a higher inlet temperature in the spray-drying process [4,60]. On the other hand, it can be seen that a higher percentage of encapsulants is associated with a significant decrease in the concentration of these compounds. This is explained by the fact that a lower amount of phenolic extracts was used in the nanocapsule formulation.

The effects of temperature and encapsulant percentage (*p* < 0.05) on flavonoid content are presented in Figure 3b. It is observed that an increase in both temperature and encapsulant percentage leads to an increase in flavonoid content. A possible explanation for the simultaneous increase in flavonoids with both variables may be related to the enhancement of protective mechanisms of the encapsulant matrix at high temperatures [4] and a significant contribution of the flavonoids present in quinoa [61].

Figure 3c shows the interaction of temperature and percentage of encapsulant (*p* < 0.05) on anthocyanin content. It can be seen that the higher the temperature, the higher the anthocyanin content, and the higher the percentage of the encapsulant, the lower the anthocyanin content. The increase in anthocyanin content with increasing drying temperatures is due to a higher stability as a result of the reduction in water activity [62,63]. However, increasing the encapsulating agent content decreased the anthocyanins due to the dilution effect of the encapsulation matrix, as it limits the diffusion of anthocyanins [64,65].

Figure 3d shows the effects of temperature and the percentage of the encapsulant (*p* < 0.05) on the antioxidant capacity by DPPH; a slight increase in the antioxidant capacity can be observed due to the effect of the higher temperature, while the higher the percentage of the encapsulant, the lower the antioxidant capacity. The increase in temperature could be attributed to the destabilization of the nanocapsule walls, facilitating the release of bioactive compounds [4]. However, increasing the concentrations of the encapsulating agents reduced the antioxidant activity, a behavior related to the decrease in the content of bioactive compounds due to the dilution effect of the matrix [60].

**Figure 4:**
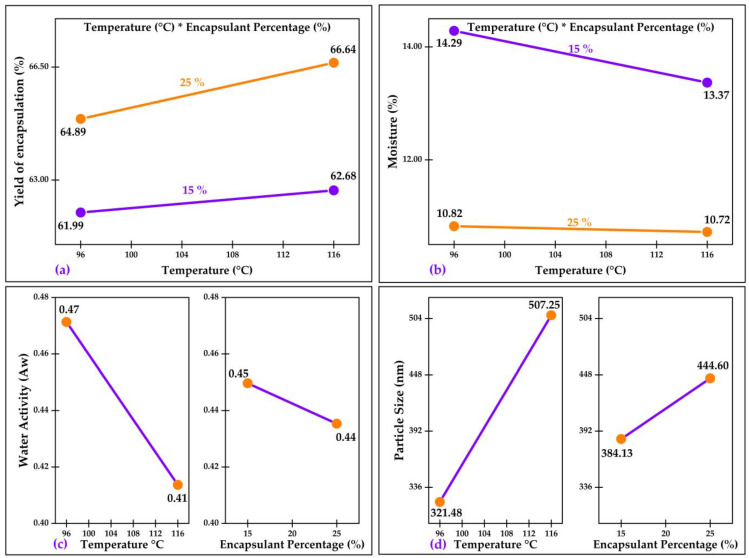
Effects and interactions in: (**a**) yield of encapsulation; (**b**) moisture; (**c**) water activity; and (**d**) particle size. * Corresponds to the multiplication sign.

Figure 4a shows the interaction of temperature and the percentage of the encapsulant (*p* < 0.05) on the encapsulation yield. It can be observed that the higher the temperature and percentage of the encapsulant, the higher the yield obtained. High spray-drying temperatures improve the encapsulation efficiency of bioactive compounds by promoting rapid formation of the capsule wall [32,60]. Likewise, a higher concentration of encapsulating material increases the retention of the active nucleus due to a greater matrix thickness [4,30].

Figure 4b shows the interaction of temperature and the percentage of the encapsulant (*p* < 0.05) on moisture. It can be seen that the higher the temperature and percentage of the encapsulant, the lower the humidity. Higher temperatures promoted greater evaporation of water from the nanocapsules [4,30]. Likewise, increasing the concentrations of encapsulating agents favors the formation of drier and more stable matrices [33,47]. Figure 4c shows the effects of temperature and the percentage of the encapsulant (*p* < 0.05) on water activity. It can be seen that the higher the temperature and percentage of the encapsulant, the lower the water activity. It is known that increasing spray-drying temperatures promotes the rapid evaporation of water, reducing the water activity in the powders obtained [60]. Also, higher concentrations of encapsulating agents can form more homogeneous and stable matrices, limiting water availability [66]. The synergistic decrease in moisture and water activity by using high temperatures and encapsulant percentages has a positive impact on the stability and shelf life of the encapsulated bioactive compounds.

Figure 4d shows the effects of temperature and the percentage of the encapsulant (*p* < 0.05) on particle size. It can be seen that the higher the temperature and percentage of encapsulant, the larger the particle size. Higher drying temperatures produced larger particle sizes by promoting rapid microcapsule wall formation. Also, increasing the concentrations of the encapsulating agents can increase the viscosity of the dispersion, forming larger droplets during spray-drying [4,59]. Particle size control is relevant to improving the flow and dispersibility properties of the obtained powders.

### 2.4. Instrumental Characterization of Nanocapsules

#### 2.4.1. Color, ζ-Potential, and SEM-EDS Analysis of Nanocapsules

**Figure 5:**
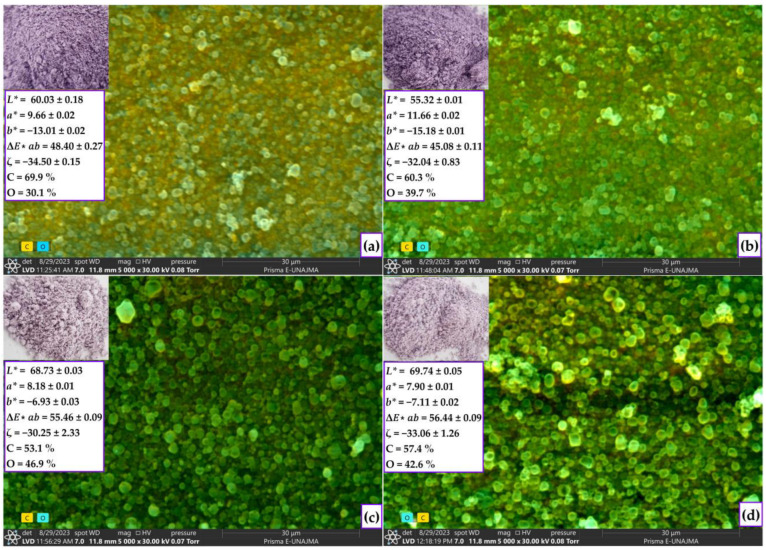
Instrumental characterization in treatments T1 (**a**); treatment T2 (**b**); treatment T3 (**c**); and treatment T4 (**d**).

Figure 5a–d shows the instrumental characterization of the nanocapsules of treatments T1 (96 °C and 15%), T2 (116 °C and 15%), T3 (96 °C and 25%) and T4 (116 °C and 25%), respectively. As for color, an increase in lightness (L*) was observed with increasing the encapsulant percentage, while there was a decrease in chroma (a*) and (b*). This behavior could have arisen because increasing the concentrations of the encapsulant material caused a dilution effect on the pigments of the encapsulated extract [4,67]. In addition, the color variation was calculated by taking, as reference, the initial value of the color of the liquid extract of the native potato (L* = 14.06, a* = 0.33, and b* = −0.61), resulting in more significant differences in the treatments with a higher percentage of the encapsulant. The values of the ζ potential varied between −30.25 and −34.50, indicating moderate stability of the particles in suspension, which is related to the electrostatic repulsions between particles [68,69]. Characterization by SEM-EDS microscopy revealed the majority presence of carbon and oxygen, and smooth, spherical, and homogeneous particles characteristic of nanoencapsulations formed by spray-drying were also observed. In addition, the small particles agglomerated around the large ones are due to electrostatic interactions [4,30,59]. Increasing the temperature and percentage of the encapsulant produced a larger particle size (treatments T2 y T4), possibly due to the higher wall formation rate and dispersion viscosity during spray-drying [4,30]. Size control is crucial to improve the physicochemical stability, modified release, and bioavailability of the nanoencapsulated system [1,70].

#### 2.4.2. FTIR Analysis

**Figure 6:**
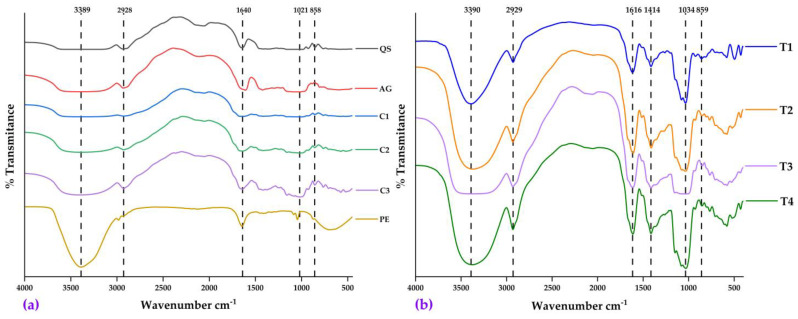
(**a**) FTIR analysis on quinoa starch (QS), arabic gum (AG), fresh Kulli papa (C1), fresh Sumaqcha (C2), fresh Zambita (C3), and phenolic extract of Kulli papa (PE); and (**b**) FTIR analysis on nanocapsules of treatments T1, T2, T3 and T4.

The Fourier transform infrared spectroscopy (FTIR) technique was used to confirm the successful encapsulation of phenolic extracts of native Kulli potato in the polymeric matrix by a validated methodology [30,32,71,72]. The FTIR spectra (Figure 6a,b) show the leading characteristic bands of both the starting materials (quinoa starch, gum arabic, fresh native potatoes, and phenolic extract of the chosen variety) and the nanocapsules obtained in the treatments of the experimental design.

In all the spectra, an intense wave number of around 3389–3390 cm^−1^ assigned to the OH stretching of the hydroxyl groups of the phenolic extract and quinoa starch is identified. Other common wavenumbers observed in both the starting materials and nanocapsules were 2928–2929 cm^−1^, associated with the CH stretching of CH_2_ and CH_3_ groups in aliphatic chains; 1616–1640 cm^−1^, related to OH bending by adsorbed water; 1414 cm^−1^, corresponding to in-plane deformation of OH groups; 1021–1034 cm^−1^, assigned to C-O tensile vibration; and 858–859 cm^−1^, attributed to aromatic CH deformation [4,30,32].

The characteristic signals of both the extract and starch in the FTIR spectra of the nanocapsules confirm the effective encapsulation in the polymeric matrix [73,74]. It was also shown that the encapsulation occurred through physical interactions; the absence of significant alterations in the relative signals and intensities corroborates a predominantly physical mechanism, coinciding with previous studies of similar systems [4,30,32,59].

#### 2.4.3. Thermal Analysis

**Figure 7:**
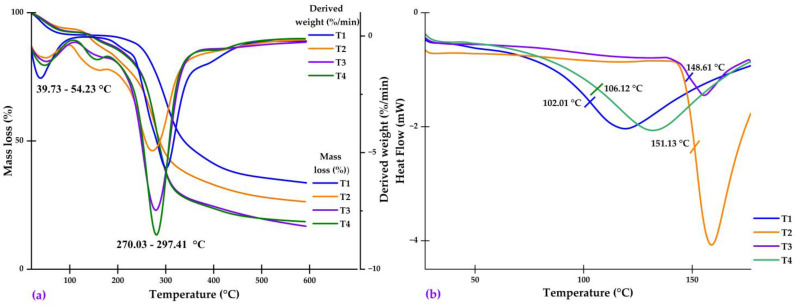
(**a**) TG and DTA analysis on nanocapsules; and (**b**) DSC analysis on nanocapsules.

The results of the thermal analysis confirm the effective encapsulation of native potato phenolic extracts in the quinoa starch and gum arabic matrix. The TG/DTA analysis showed two main mass loss events (Figure 7a), attributed to the removal of water/volatile compounds starting between 39.73–54.23 °C and then to the decomposition of biopolymers such as carbohydrates, proteins, and fiber between 270.03–297.41 °C. The temperature of these events agrees with previous studies on phenolic extract encapsulates [4,30,32]. DSC analysis revealed glass transition temperatures between 102.01–151.13 °C (Figure 7b), expected values considering the Tg reported in the present investigation for quinoa starch, gum arabic, and dehydrated phenolic extract. Multiple Tg indicates the formation of a heterogeneous matrix due to interactions between encapsulating biopolymers and bioactive compounds [46,75]. Glass transition temperatures are related to the chemical composition, molecular weight, and moisture. Fourier transform infrared spectroscopy and DSC are used to analyze the interactions between polyphenol-rich extracts and the matrix. The endothermic peaks in the DSC analysis reflect the glass transition and melting attributed to the polysaccharides used as encapsulating materials [4,58]. Taken together, these results demonstrate that spray-drying effectively encapsulated native potato phenolic extracts, forming stable nanocapsules with potential applications in food and pharmaceutical systems.

#### 2.4.4. Release of Phenolic Compounds in the Nanocapsules

**Figure 8:**
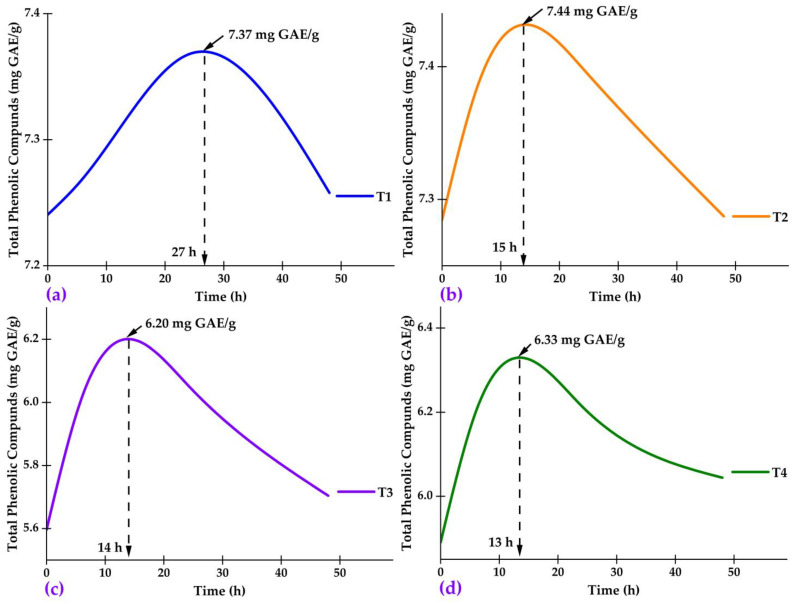
Release of phenolic compounds in the nanocapsules: (**a**) Treatment T1; (**b**) Treatment T2; (**c**) Treatment T3; and (**d**) Treatment T4.

The release was evaluated by measuring total phenolic compounds as a function of time (Figure 8a–d). The results showed that the drying temperature and the proportion of encapsulating agents affected the release kinetics of the phenolic compounds. In the T1 treatment (96 °C and 15% encapsulant), a continuous release of phenolic compounds was observed until reaching 7.37 mg GAE/g at 24 h. The lower drying temperature in this treatment could have contributed to a slower drying rate, resulting in a matrix with a less dense polymeric network, allowing gradual diffusion of the phenolic compounds [76,77,78]. On the other hand, in the T2 treatment (116 °C and 15% encapsulant), a maximum release content of 7.44 mg GAE/g was reached at 15 h, indicating a positive effect of the higher drying temperature on the release kinetics. This may be attributed to forming a more porous polymeric matrix due to the faster evaporation of water at higher temperatures [30,79]. In the treatments with a higher proportion of encapsulating agents, T3 (96 °C and 25%) and T4 (116 °C and 25%), a lower amount of released phenols was observed (6.20 and 6.33 mg GAE/g, respectively). This is probably due to the formation of a denser polymeric network with lower porosity due to the higher amount of starch and gum in the formulation, restricting the diffusion of phenolic compounds [4,32,80,81,82]. In conclusion, the drying temperature and the proportion of encapsulating agents significantly affected the kinetics and release capacity of phenolic compounds from the nanocapsules, allowing them to modulate the release according to the application needs. Several innovative techniques are currently being explored, such as 3D printing, microfluidization (which uses high pressures to produce uniform and small nanocapsules), coacervation (which induces spontaneous nanocapsule formation by adjusting pH, temperature, and ionicity), and ultrasound (which improves encapsulation efficiency through acoustic cavitation). Freeze-drying technologies, such as freeze-drying, benefit thermosensitive compounds and create porous structures to modulate release.

## 3. Materials and Methods

### 3.1. Materials

**Figure 9:**
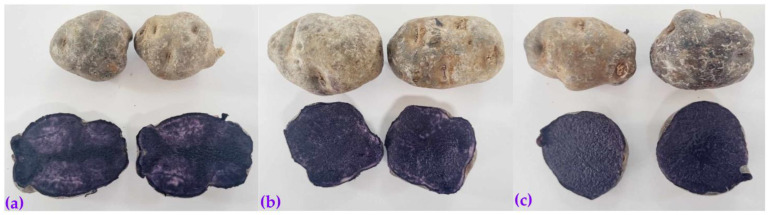
Colored native potatoes: (**a**) Kulli papa; (**b**) Sumaqcha; and (**c**) Zambita.

Samples of native potatoes (*Solanum tuberosum* spp. *andigena*), known as Kulli papa, Sumaqcha, and Zambita (Figure 9), were obtained from the company “SEMPAL S.R.L.” in the district of San Jeronimo, province of Andahuaylas, Apurimac Region, Peru. Quinoa (*Chenopodium quinoa*) of the white Junín variety was purchased at the central market in the province of Andahuaylas. A commercial sample of *Acacia senegal* gum arabic of the Fratello brand produced by Importaciones Goicochea S.A.C. in Peru was also purchased.

In the case of native potato crops, an average yield of 20 t/ha was achieved. For the extraction of bioactive compounds, tubers of the third category (31–60 g and 71–90 mm) and fourth category (less than 30 g and less than 70 mm), which are considered as discarded by producers, were used. Tubers were selected on the basis of their high field yields and bioactive compound content. Also, factors such as parent generation, geographic location, and the number of experiments used were considered.

Analytical-grade reagents and consumables were appropriately utilized in the laboratory. The following chemicals were used in the experiment: ethanol (Scharlau, Senmanat, Spain), citric acid (Frutarom, Lima, Peru), gallic acid (Merck, Darmstadt, Germany), Folin–Ciocalteu reagent (Himedia, Dindori, India), methanol (J.T. Baker, Mexico City, Mexico), quercetin standard (Sigma Aldrich, St. Louis, MO, USA), Aluminum chloride (Sigma Aldrich, St. Louis, MO, USA), potassium chloride (Spectrum, New Brunswick, New Brunswick, USA), sodium acetate trihydrate (Spectrum, New Brunswick, New Brunswick), hydrochloric acid (Spectrum, New Brunswick, Canada), sodium carbonate (Spectrum, New Brunswick, Canada), Trolox standard (Sigma Aldrich, St. Louis, MO, USA), DPPH reagent (HIMEDIA, Mumbai, India), ABTS reagent (Sigma Aldrich, St. Louis, MO, USA), potassium persulfate (Biolab, Buenos Aires, Argentina), IR grade potassium bromide (Thermo Fisher Scientific, Garfield, NJ, USA).

### 3.2. Quinoa Starch

A total of 2 kg of quinoa of the Blanca Junín variety were weighed and washed. Then, they were cut into small pieces and crushed using a blender (Silentmixx Pro, Bosch, Stuttgart, Germany). The mixture was left to stand under refrigeration for 12 h. Then, successive washes were performed in a centrifuge (TDL-5M, Bioridge, Shanghai, China) at 3500 RPM for 1 min to eliminate the fiber. Then, the mixture was dried at a temperature of 40 °C in a forced convection oven FED 115 (BINDER, Tuttlingen, Germany) for 24 h. An agate mortar was employed for the grinding process, followed by a sieving procedure using a 63 µm mesh on an analytical sieve shaker (AS 200, Retsch, Haan, Germany) [48,83,84].

### 3.3. Extraction of Phenolic Compounds

A mixture of 96% ethanol and water acidified with citric acid at a pH of 3, at a ratio of 85:15, was used to extract phenolic compounds. A total of 50 g of native potato that had been mashed beforehand was used, along with 100 milliliters of an ethanol–water solution. This mixture was left to agitate for 24 h at room temperature at a speed of 250 RPM. Afterward, the obtained extract was filtered using Whatman 41° filter paper (Whatman International LTD, Maidstone, UK). It was then placed in a vacuum oven (model VD56, Binder, Tuttlinger, Germany) at a pressure of 10 mbar and a temperature of 25 °C in order to remove the excess ethanol. The resulting extract was refrigerated in an amber container until further use [4,85].

### 3.4. Nanoencapsulation of Phenolic Compounds

**Figure 10:**
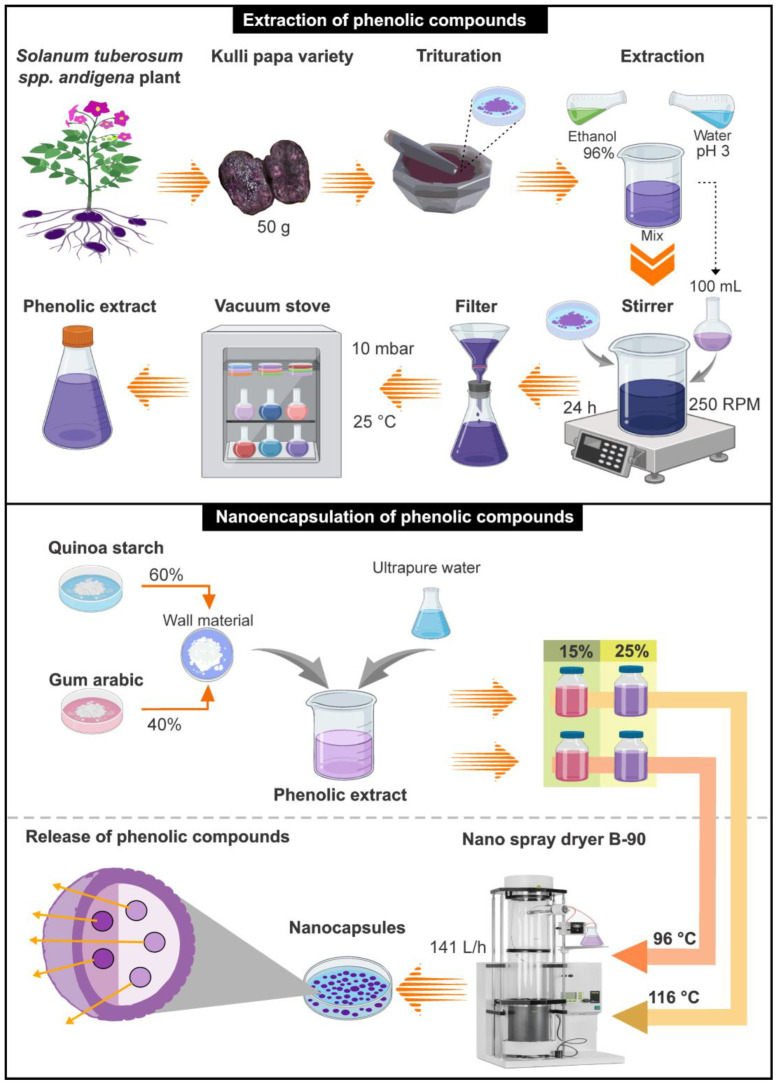
Experimental flow diagram.

A mixture of 60% quinoa starch and 40% gum arabic (*w*/*w*) was utilized as the encapsulation material. A total of 10 mL of the phenolic extract was measured, and then 10 mL of 15% and 25% (*w*/*v*) of the encapsulating solutions were introduced, making up the total volume to 80 mL using ultrapure water. A 2^2^ factorial design was used for the nanoencapsulation, considering temperature (96 and 116 °C) and the percentages of the encapsulant (15% and 25%) as independent variables. A B-90 nano spray-dryer (BÜCHI Labortechnik AG, Flawil, Switzerland) was used at an airflow rate of 141 L/h, with 100% suction, using the 4 µm nebulizer. Previous nanoencapsulation research work developed by the authors allowed for the establishment of the present study’s inlet temperatures and encapsulant percentages. [4,30]. The experimental flow diagram is shown in Figure 10.

### 3.5. Phenolic Compounds

A total of 1g of the sample and 20 mL of methanol containing 80% of the concentration were utilized for creating methanolic extracts. These extracts were subjected to a 24 h maceration process at room temperature while being shielded from light. Total phenolic compounds were quantified using the Folin–Ciocalteu method, with gallic acid employed for the calibration curve. A total of 0.9 mL of the methanolic extract was taken, along with 2.4 mL of ultrapure water (considering a dilution factor of 3.7). Additionally, 0.15 mL of 20% Na_2_CO_3_ and 0.3 mL of 0.25N Folin–Ciocalteau reagent were also added. After a quarter of an hour, the absorbance values were measured using a UV spectrophotometer at a wavelength of 755 nm. (CR-5, Konica Minolta, Tokyo, Japan) [32,86,87,88].

### 3.6. Flavonoids

A quercetin solution in ethanol was utilized as the standard for preparing the calibration curve. In the extraction of flavonoids, 0.5 g of the sample was combined with 20 mL of 80% methanol, maintaining it at room temperature and shielding it from light for 24 h. To quantify flavonoids, 90 µL of the extract, 100 µL of AlCl_3_, and 4.81 mL of methanol at an 80% concentration were utilized. Measurements were recorded at a wavelength of 425 nm using a UV spectrophotometer [4,89].

### 3.7. Anthocyanins

The pH differential method introduced by Giusti and Wrolstad was employed. Ethanolic extracts were created by combining 20 mL of a solution containing 95% ethanol and 1% HCl with 1 g of the sample. This mixture was allowed to react for 24 h. Subsequently, the samples were treated with 0.025 M KCl and 0.4 M C_2_H_3_NaO_2_ buffer solutions, adjusting the pH levels to 1 and 4.5, respectively. Readings were taken at a wavelength of 700 nm using a UV spectrophotometer (Genesys 150, Thermo Fisher Scientific, Waltham, MA, USA). The previously determined dilution factor with the KCl buffer was taken into account. The outcomes were expressed in milligrams of anthocyanin per gram of sample on a dry weight basis [45,90].

### 3.8. Antioxidant Capacity by DPPH

Methanolic extracts were produced by combining 0.5 g of the sample with 20 mL of methanol at an 80% concentration, allowing the mixture to stand at room temperature for 24 h. A calibration curve was established using Trolox (6-hydroxy-2,5,7,8-tetramethylchroman-2-carboxylic acid). To create a diluted solution of DPPH (2,2-diphenyl-1-picrylhydrazyl), free radicals with an absorbance of 1.1 ± 0.02 at 515 nm and 150 µL of the sample extract were mixed with 2850 µL of the diluted DPPH solution and left to react for 15 min at room temperature in light-protected test tubes. Simultaneously, a blank was prepared using 150 µL of methanol. Readings were taken at 515 nm and expressed as µmol of Trolox equivalents per gram of dry sample [45,86,87,88].

### 3.9. Antioxidant Capacity by ABTS

The ABTS+ radical was generated by mixing 250 µL of 2.45 mmol K_2_S_2_O_8_ with 25 mL of 7 mmol ABTS and letting it undergo a 16 h reaction at room temperature in darkness. The absorbance of the ABTS+ solution was adjusted to 0.7 ± 0.02 at a wavelength of 734 nm. Subsequently, 300 µL of the methanolic extract was combined with 2700 µL of ABTS+ and allowed to react for 15 min. Methanol was used to prepare a control sample consisting of 300 µL. Measurements were taken at 734 nm, and the results were expressed in µmol ET/g of dry sample [30,87].

### 3.10. Water Activity

A HygroPalm23-AW portable water activity meter (Rotronic, Bassersdorf, Switzerland) was used and adequately calibrated. Readings were taken at a temperature of 25 °C [33,91].

### 3.11. Moisture

The determination was carried out following the AOAC 950.10 methodology. A quantity of 2 g of the sample was carefully weighed and positioned in watch glasses, then placed inside a forced convection oven, model FED 115 (BINDER in Tuttlingen, Germany). The oven maintained a temperature of 105 °C until a consistent weight was attained. Each sample’s initial and final mass values were recorded for the subsequent calculation [91,92].

### 3.12. Particle Size

Particle size analysis was conducted using dynamic light scattering (DLS) employing a ZU3100 Zetasizer (Malvern Instruments in Worcestershire, UK). For sample preparation, 20 mg of nanocapsules were dispersed in ultrapure water and subjected to ultrasound for a duration of 10 s. Readings were carried out at 20 °C using a 633 nm He–Ne laser in a DTS002 cell [4,93].

### 3.13. Color Analysis

Colorimetric data were obtained from the samples through the reflectance accessory of the CR-5 colorimeter (Konica Minolta, Tokyo, Japan) using a high-precision cell of 30 mm diameter [47,91].

### 3.14. ζ Potential

A Zetasizer ZSU3100 (Malvern Instruments, Worcestershire, UK) equipped with a He–Ne laser was used to calculate the ζ-potential. A total of 20 mg of the nanocapsules were dispersed in 50 mL of ultrapure water and underwent ultrasound treatment for 60 s. The measurements were taken at a temperature of 25 °C within a DTS1080 cell [32,94].

### 3.15. Amylose and Amylopectin

A calibration curve was established using concentrations ranging from 0.1 to 1.0 mg/mL, utilizing a standard of potato amylose. A total of 20 mg of the sample was utilized; 200 µL of 95% ethanol and 1800 µL of 1 M NaOH were added, allowing them to sit for 24 h at room temperature. The volume was subsequently adjusted to 20 mL with ultrapure water and homogenized at 2000 RPM. For the colorimetric reaction, 500 µL of the extracted solution, 1 mL of 1 M acetic acid, and 200 µL of lugol solution were taken, and the volume was brought up to 10 mL with ultrapure water. The solution was agitated and left to react for 20 min, carefully shielding it from light. Absorbance readings were taken at a wavelength of 620 nm using a UV spectrophotometer (CR-5, Konica Minolta, Tokyo, Japan) [47,95].

### 3.16. SEM-EDS Analysis

The morphological analysis was performed using a scanning electron microscope (Prisma E, Thermo Fisher Scientific, Brno, Czech Republic). The specimens were arranged on 12 mm adhesive carbon strips. The examination was conducted in low-vacuum mode, employing ABS and LVD detectors at a pressure of 0.07 Torr [4,96].

### 3.17. FTIR Analysis

FTIR analysis was conducted using the Nicolet IS50 FTIR transmission module (ThermoFisher in Waltham, MA, USA). Pellets were prepared by combining 2 mg of the sample with 200 mg of KBr and then pressed at 10 tons of force for 30 s. Spectra were acquired within the mid-IR range, employing the KBr beam splitter at a resolution of 8 cm^−1^, with 32 scans [97,98].

### 3.18. Thermal Analysis

A total of 10 mg of the nanocapsules were employed for thermogravimetric analysis (TGA 550, TA Instruments, New Castle, DE, USA). This analysis encompassed a temperature span from 20 to 600 °C, with a heating rate of 10 °C per minute. The entire procedure took place in a nitrogen gas atmosphere [99].

A total of 2 mg of the nanocapsules were employed for conducting the differential scanning calorimetry analysis (DSC2500, TA Instruments, New Castle, DE, USA). The temperature range extended from 0 to 250 °C, with a heating rate of 10 degrees per minute, and the procedure took place in a nitrogen-rich environment [47,100].

### 3.19. Release of Phenolic Compounds

Solutions were made by dissolving 100 mg of the nanocapsules in 10 mL of ultrapure water. These solutions were left at room temperature, shielded from light. Readings were taken after 0, 6, 24, and 48 h using a UV spectrophotometer at a wavelength of 755 nm [32].

### 3.20. Statistical Analysis

The experiment included an analysis of variance, followed by identifying significant differences using Tukey’s multiple range test at a significance level of 5%. Visual representation and statistical tests were conducted using Origin Pro 2023 (OriginLab Corporation, Northampton, MA, USA).

## 4. Conclusions

The inlet temperature and the percentage of quinoa starch/gum arabic significantly affected the properties of nanocapsules obtained by spray-drying from native potato extracts. It was found that a high drying temperature (116 °C) improved the retention of thermolabile bioactive compounds, such as phenols, flavonoids, and anthocyanins, as well as the antioxidant capacity of the nanocapsules. However, a high concentration of quinoa starch/ gum arabic encapsulant (25%) caused a dilution of the encapsulated phenolic compounds and anthocyanins. The interaction between the temperature and encapsulant percentage allowed for the modulation of critical properties such as moisture, water activity, yield, and release of the encapsulated compounds. The best conditions for the nanoencapsulation of thermolabile compounds from native potatoes were an inlet temperature of 116 °C and 15% quinoa starch/gum arabic as the encapsulating agent. The nanocapsules developed present applicability for incorporation in functional foods and pharmaceutical products. The knowledge obtained will positively impact local, regional, and global levels to solve technological problems in nanoencapsulation and the use of undervalued Andean crops.

## Figures and Tables

**Table 1:** Physical and chemical properties of fresh native potatoes.

Properties	Kulli Papa				Sumaqcha				Zambita			
	$\bar{x}$	±	SD	*	$\bar{x}$	±	SD	*	$\bar{x}$	±	SD	*
Phenolic Compounds (mg GAE/g)	3.90	±	0.01	a	2.84	±	0.01	b	3.11	±	0.01	c
Anthocyanins (mg C3G/g)	401.22	±	1.78	a	135.53	±	1.88	b	196.62	±	1.01	c
Flavonoids (mg of QE/g)	0.74	±	0.03	a	0.46	±	0.06	b	0.68	±	0.04	a
Antioxidant Capacity DPPH (µmol ET/g)	23.43	±	0.05	a	4.35	±	0.12	b	6.95	±	0.07	c
Antioxidant Capacity ABTS (µmol ET/g)	19.22	±	0.04	a	9.99	±	0.04	b	10.73	±	0.11	c
*L*	13.55	±	0.24	a	14.89	±	0.22	b	22.15	±	0.15	c
*a*	4.24	±	0.11	a	6.54	±	0.15	b	3.63	±	0.14	c
*b*	−5.45	±	0.02	a	−5.51	±	0.13	a	−5.37	±	0.14	a
Aw	0.84	±	0.00	a	0.83	±	0.00	b	0.82	±	0.00	c
Moisture (%)	76.73	±	1.66	a	76.44	±	0.03	a	75.92	±	0.26	a

Where $\bar{x}$ is the arithmetic mean and SD is the standard deviation. * Different letters indicate significant difference per row evaluated through at 5% significance, for *n* = 3.

**Table 2:** Physical and chemical properties in nanocapsules.

Run	A	B	Phenolics Compounds	Flavonoids	Anthocyanins	AC DPPH	Yield	Moisture	Aw	Particle Size
	°C	%	mg GAE/g	mg Quercetin/g	mg C3G/g	µmol TE/g	%	%		nm
			$\bar{x}$ ± SD	$\bar{x}$ ± SD	$\bar{x}$ ± SD	$\bar{x}$ ± SD	$\bar{x}$ ± SD	$\bar{x}$ ± SD	$\bar{x}$ ± SD	$\bar{x}$ ± SD
T1	96	15	5.49 ± 0.02	0.77 ± 0.08	1.89 ± 0.02	181.06 ± 1.49	61.99 ± 0.27	14.29 ± 0.62	0.48 ± 0.01	289.57 ± 1.94
T2	116	15	5.50 ± 0.06	0.87 ± 0.10	2.32 ± 0.02	185.50 ± 1.94	62.68 ± 0.61	13.37 ± 0.06	0.42 ± 0.01	478.70 ± 1.01
T3	96	25	4.61 ± 0.16	0.63 ± 0.26	0.91 ± 0.05	48.98 ± 1.51	64.89 ± 1.05	10.82 ± 0.08	0.46 ± 0.02	353.40 ± 1.68
T4	116	25	4.89 ± 0.21	0.64 ± 0.22	0.92 ± 0.12	49.15 ± 1.21	66.64 ± 0.95	10.72 ± 0.09	0.41 ± 0.01	535.80 ± 1.36

Where A is the inlet temperature and B is the percentage encapsulant, $\bar{x}$ is the arithmetic mean and SD is the standard deviation. for *n* = 3.

## Data Availability

Data are available in the same article.
